# Role of CXCR3/CXCL10 Axis in Immune Cell Recruitment into the Small Intestine in Celiac Disease

**DOI:** 10.1371/journal.pone.0089068

**Published:** 2014-02-20

**Authors:** Constanza Bondar, Romina E. Araya, Luciana Guzman, Eduardo Cueto Rua, Nestor Chopita, Fernando G. Chirdo

**Affiliations:** 1 Laboratorio de Investigación en el Sistema Inmune – LISIN, Facultad de Ciencias Exactas, Universidad Nacional de La Plata, La Plata, Argentina; 2 Servicio de Gastroenterología, Hospital de Niños “Sor María Ludovica”, La Plata, Argentina; 3 Servicio de Gastroenterología, Hospital San Martín, La Plata, Argentina; Instutite of Agrochemistry and Food Technology, Spain

## Abstract

Lymphocytic infiltration in the *lamina propria* (LP), which is primarily composed of CD4^+^ Th1 cells and plasma cells, and increased numbers of intraepithelial lymphocytes (IELs), is a characteristic finding in active celiac disease (CD). Signals for this selective cell recruitment have not been fully established. CXCR3 and its ligands, particularly CXCL10, have been suggested to be one of the most relevant pathways in the attraction of cells into inflamed tissues. In addition, CXCR3 is characteristically expressed by Th1 cells. The aim of this work was to investigate the participation of the chemokine CXCL10/CXCR3 axis in CD pathogenesis. A higher concentration of CXCL10 was found in the serum of untreated CD patients. The mRNA levels of CXCL10 and CXCL11 but not CXCL9 were significantly higher in duodenal biopsies from untreated CD patients compared with non-CD controls or treated patients. The results demonstrate that CXCL10 is abundantly produced in untreated CD and reduced in treated patients, and the expression of CXCL10 was found to be correlated with the IFNγ levels in the tissue. Plasma cells and enterocytes were identified as CXCL10-producing cells. Moreover, the CXCL10 expression in intestinal tissues was upregulated by poly I:C and IL-15. IELs, LP T lymphocytes, and plasma cells, which infiltrate the intestinal mucosa in untreated CD, express CXCR3. The CXCR3/CXCL10 signalling axis is overactivated in the small intestinal mucosa in untreated patients, and this finding explains the specific recruitment of the major cell populations that infiltrate the epithelium and the LP in CD.

## Introduction

Celiac disease (CD) is an immune-mediated enteropathy caused by ingested gluten in genetically susceptible individuals. Active CD is characterised by histological changes in the small intestinal mucosa, such as villous atrophy, crypt hyperplasia, infiltration of lymphocytes, primarily T cells and plasma cells, into the *lamina propria* (LP), and increased intraepithelial lymphocytes (IELs). Mechanisms of both innate and adaptive immunity participate in intestinal mucosal damage, which involves disruption of tight junction integrity and the production of proinflammatory cytokines, during the early phase of CD. Direct damage to epithelial cells is considered to be primarily caused by the infiltration and activation of IELs, and IL-15 is hypothesised to play a major role by favouring the survival and cytotoxicity of these cells [1]. It has been clearly established that gluten peptides activate HLA-DQ2- or DQ8-restricted-CD4^+^ T lymphocytes. These T cells belong to the Th1 subset and, upon activation, produce high amounts of IFNγ [2]. This abundance of LP Th1 cells is largely responsible for the maintenance of an appropriate environment for the cytotoxic activity of IELs and for antibody production at the duodenal mucosa in untreated CD patients [3].

Antigen-loaded dendritic cells migrate out of the LP to the mesenteric lymph nodes, where these dendritic cells activate and differentiate naïve CD4^+^ T cells into Th1 cells. Upon differentiation, Th1 cells circulate in the peripheral blood and finally arrive into the LP under guidance by MadCAM1/α4β7 and CCL25/CCR9. These pairs of ligand/receptors are involved in the selective migration of lymphocytes into the intestinal mucosa under homeostatic conditions [4]. However, during an inflammatory process, cell recruitment is preferentially guided by other pathways. CXCR3 and its ligands have been suggested to be one of the most relevant chemokine axes that promote the arrival of cells into inflamed tissues [5]. This axis is known to be active in different chronic inflammatory processes, such as rheumatoid arthritis [6], [7] and inflammatory bowel diseases [8]–[10]. CXCR3 is expressed in T and B lymphocytes, NK cells, eosinophils, and monocytes [11]. In particular, CD4^+^ Th1 cells characteristically express CXCR3 [5], [12], [13]. This receptor interacts with three ligands: CXCL9, CXCL10, and CXCL11. These chemokines have non-redundant biological roles. All of these chemokines are inducible by IFNγ; however, their pattern of expression in different tissues has not been fully elucidated [14]. Of the CXCR3 ligands, CXCL10 shows a strong association with autoimmunity [6], [15], [16]. CXCL10 is produced by CD4^+^ T cells, NK and NKT cells, monocytes, dendritic cells, fibroblasts, endothelial, and epithelial cells [7], [17]. In addition to IFNγ, other stimuli, such as TNFα and type I IFN, induce CXCL10 expression and thereby amplify the inflammatory cascade [14].

Although the infiltration of lymphocytes into the LP and increased IELs are hallmarks in CD enteropathy, the mechanism underlying specific cell recruitment has not been studied. Because Th1 cells characteristically express CXCR3 and certainly take part in the damage mechanisms that cause severe enteropathy, the aim of this work was to assess the role of the CXCL10/CXCR3 axis in lymphocytic recruitment in active CD. In this study, we demonstrate the increased production of CXCL10 in the epithelium and LP, primarily by enterocytes and plasma cells, respectively, in the intestinal mucosa of untreated CD patients. CXCL10 was induced in the duodenal tissue by innate stimuli. We also showed the expression of CXCR3 in IELs and in LP T lymphocytes and plasma cells.

## Patients and Methods

### Samples

Duodenal biopsies were obtained from paediatric and adult patients during routine procedures to diagnose celiac disease. In total, 26 untreated celiac patients (six adults and 20 children) and six treated CD patients (three adults and three children) were included in the gene expression analysis. CD diagnosis was achieved by histological examination, serological analysis, and the evaluation of clinical presentation. Patients on a gluten-free diet (GFD) presented histological recovery and negative serological markers for CD. Twenty-five biopsies from non-celiac individuals (nine adults and 16 children) were also included in this study. All of the individuals who suffered from other gastrointestinal conditions, primarily dyspepsia, presented negative CD serology and normal duodenal histology. The samples were stabilised using RNAlater (Ambion, cat AM7020) and stored at −80°C until processing for gene expression analysis. An additional biopsy was obtained, formalin fixed and embedded in paraffin for immunofluorescence studies. For some experiments, two biopsy pieces were collected for *in vitro* stimulation, as described below. In total, 23 untreated celiac patients and 32 non-CD controls were included in this assay.

Serum samples were collected from 26 celiac patients at the time of diagnosis (15 adults and 11 children), nine treated CD patients (six adults and three children), and 21 control subjects (14 adults and seven children). Because there was no difference between the samples obtained from paediatric and adult populations, the data from the samples from both populations were depicted together in all of the analyses that were performed in this study.

### Ethics Statements

The participants or their guardians provided written informed consent to participate in this study. The present study was approved by the Ethical Committees of the HIGA San Martin and Sor Maria Ludovica Hospitals from La Plata, Buenos Aires, Argentina.

### Gene Expression Analysis

The total RNA was isolated from whole biopsy samples using an RNA Spin Mini kit (GE Healthcare, cat 25-0500-72). The RNA quality and quantity were assessed through conventional spectrophotometric methods. Reverse transcription was performed using 1 µg of the total RNA. MML-V polymerase and random primers were obtained from Molecular Probes Inc., Invitrogen (Carlsbad, CA, USA). Real-time PCR was performed using an IQ-Cycler (Bio-Rad) with the SYBR Green Supermix (Invitrogen, cat 11761-100). β-actin was used as the housekeeping gene. Relative quantitation of gene expression was calculated using the accurate Ct (threshold cycle) method [18]. Table 1 shows the primer pairs used in this work. The running protocol for the detection of CXCL9, CXCL10, CXCL11, and CXCR3 was the following: 95°C for 10 min and 50 cycles of 60°C for 15 s, 72°C for 45 s, and 95°C for 15 s. For IFNγ, TNFα, and IFNβ, the protocol was the following: 95°C 10 min and 50 cycles of 62°C for 1 min and 95°C 15 s.

**Table 1:** Primer pairs used for real-time PCR (5′–3′orientation).

GENE	Forward Primer	Reverse Primer
**β-actin**	ATGGGTCAGAAGTCCTATGTG	CTTCATGAGGTAGTCAGTCAGGTC
**CXCL9**	CCAAGGGACTATCCACCTACAATC	GGTTTAGACATGTTTGAACTCCATTC
**CXCL10**	CTGACTCTAAGTGGCATTCAAGGA	CAATGATCTCAACACGTGGACAA
**CXCL11**	GGGTACATTATGGAGGCTTTCTCA	GAGGACGCTGTCTTTGCATAGG
**CXCR3**	AGCTTTGACCGCTACCTGAA	TGTGGGAAGTTGTATTGGCA
**IFNγ**	GCAGAGCCAAATTGTCTCCT	ATGCTCTTCGACCTCGAAAC
**TNFα**	CTCAGCCTCTTCTCCTTCCT	TTCGAGAAGATGATCTGACTGC
**IFNβ**	TGGGAGGCTTGAATACTGCCTCAA	TCTCATAGATGGTCAATGCGGCGT

### Immunofluorescence Confocal Microscopy

Paraffin-embedded duodenal biopsies from adult celiac patients, including both treated and active patients, and controls were used. Sections of 5 µm were rehydrated and treated with Antigen Retrieval Citra Plus Solution (BioGenex, cat HK080-9K). All of the sections were blocked with goat serum. For CXCR3 staining, the sections were incubated with 15 µg/ml mouse anti-human CXCR3 antibody (R&D Systems, cat MAB160) and then with a 1∶200 dilution of Alexa Fluor 488-conjugated F(ab’)_2_ fragment of goat anti-mouse IgG (H+L) antibody (Invitrogen, cat A11020). For CXCL10 single staining, the samples were incubated with a 1∶50 dilution of polyclonal rabbit anti-human CXCL10 (IP-10) antibody (Santa Cruz, cat sc-28877) and then with a 1∶200 dilution of Alexa Fluor 488-conjugated goat anti-rabbit IgG (H+L) (Invitrogen, cat A11008). All of the antibodies were incubated for one h, and the samples were washed in PBS supplemented with 0.1% Tween-20 between each incubation step.

In the double staining protocols, the samples were first incubated with CXCL10 primary antibody and then with a 1∶200 dilution of Alexa Fluor 647-conjugated goat anti-rabbit IgG antibody (H+L) (Invitrogen, cat A21246). Mouse anti-human CD3 (DBS, cat Mob112-05), mouse anti-human CD138 (DAKO, cat *M7228*), or mouse anti-human HAM56 (Genetex, GTX72010) antibodies were used at dilutions of 1∶10, 1∶25, and 1∶50, respectively, for overnight incubations. Alexa Fluor 488-conjugated goat anti-mouse antibody was used as a secondary antibody for these markers. The nuclei were stained with propidium iodide (1 µg/ml) for 15 min (Sigma, cat P4170). The samples were mounted using fluorescent mounting medium (DakoCytomation, cat S3023) and visualised in a TCS SP5 Leica confocal microscope. The images were obtained using the Leica LAS AF software.

### Counting of CXCR3^+^ Cells

Duodenal biopsies from six adult celiac patients, nine adult controls, and six treated patients were stained for CXCR3 as previously described. The nuclei were stained with DAPI (Sigma, cat D8417), and the samples were visualised using a Nikon Eclipse Ti fluorescence microscope with an X-Cites Series 120 Q light source. The images were obtained with a Nikon Digital Sight DS Ri1 camera using the Nis-Elements software, and the cells were then counted using the Image J software, which was properly calibrated for measuring the areas. The LP areas were drawn over the entire histological section, and the average positive cells were then counted in an area of 150,000 µm^2^. The surface epithelium, villi, and crypts were excluded. The cells counts were performed blindly by the same investigator.

### Determination of CXCL10 in Serum

The CXCL10 concentration in the serum samples was measured using a commercially available enzyme-linked immunosorbent assay (ELISA kit Human IP-10 (CXCL10), cat KAC2361, Invitrogen, Camarillo, USA) following the manufacturer’s instructions. Blood samples were collected from paediatric and adult patients during the routine procedure to diagnose celiac disease. The serum samples were stored at −80°C until use in subsequent assays. Each sample with a high CXCL10 concentration was diluted 1∶2 and/or 1∶10 prior testing.

### *In vitro* Stimulation of Duodenal Tissue

Two duodenum biopsy specimens were collected from the same patient during an upper-gastrointestinal endoscopy and immediately processed. RPMI medium supplemented with 62.4 µg/ml penicillin (Bagó Laboratories), 100 µg/ml streptomycin (Bagó Laboratories), 0.5 g/l gentamicin, and 10% foetal calf serum (Gibco) was used. The samples were incubated for 3 h at 37°C in medium alone or in medium supplemented with one of the following stimuli: 50 ng/ml human recombinant IL-15 (BD Pharmingen) or 100 µg/ml polyinosinic-polycytidylic acid sodium salt (poly I:C) (Sigma Aldrich, cat P1530). After culture, the samples were washed with 0.5 g/l HBSS/gentamicin, and the total RNA was extracted. Gene expression was analysed as indicated above.

### Flow Cytometry Analysis

Two duodenal biopsies from paediatric CD patients were collected in ice-chilled RPMI medium and processed further in the laboratory within 30 min. Preparations of single-cell suspensions were performed as follows: epithelial cells and IELs were removed by incubation with 1 mM EDTA in PBS for 30 min with continuous rotation at 37°C. LP single-cell suspensions were obtained by digesting the remaining material with DNase I (Roche) and collagenase (Sigma) for 30 min with rotation at 37°C. The cell suspension was then filtered through a 40-µm cell strainer and washed with PBS. The cells were blocked with human serum and stained with labelled antibodies on ice for 15 min. After incubation, the cells were briefly washed, resuspended in PBS, and analysed with a FACSCalibur instrument (BD Biosciences). The data were analysed using the FlowJo 7.6.2 software. The following antibodies were used (all from BD Pharmingen): APC-conjugated mouse anti-human CD183 (clone 1C6/CXCR3), FITC-conjugated mouse anti-human CD3 (clone UCHT1), PE-conjugated mouse anti-human CD103 (clone Ber-ACT8), PE-conjugated mouse anti-human CD4 (clone RPA-T4), PE-conjugated mouse anti-human CD138 (clone MI15), and APC-, FITC-, or PE-conjugated mouse IgG1 κ isotype controls (clone MOPC-21).

### Statistical Analysis

The statistical analyses were performed with the Prism v.5.0 software (GraphPad software Inc., La Jolla, CA, USA), and this software was also used to construct the graphs. Two-tailed P-values less than 0.05 were considered significant. A comparison of the expression levels and the positive cell numbers between subjects, which included active and treated CD patients, was performed using unpaired t-tests. Correlation analyses were performed using Pearson’s correlation test. Statistical analysis in experiments using *in vitro* stimulated biopsies were evaluated through paired t-tests.

## Results

### Serum Levels of CXCL10 are Increased in Untreated CD Patients

**Figure 1:**
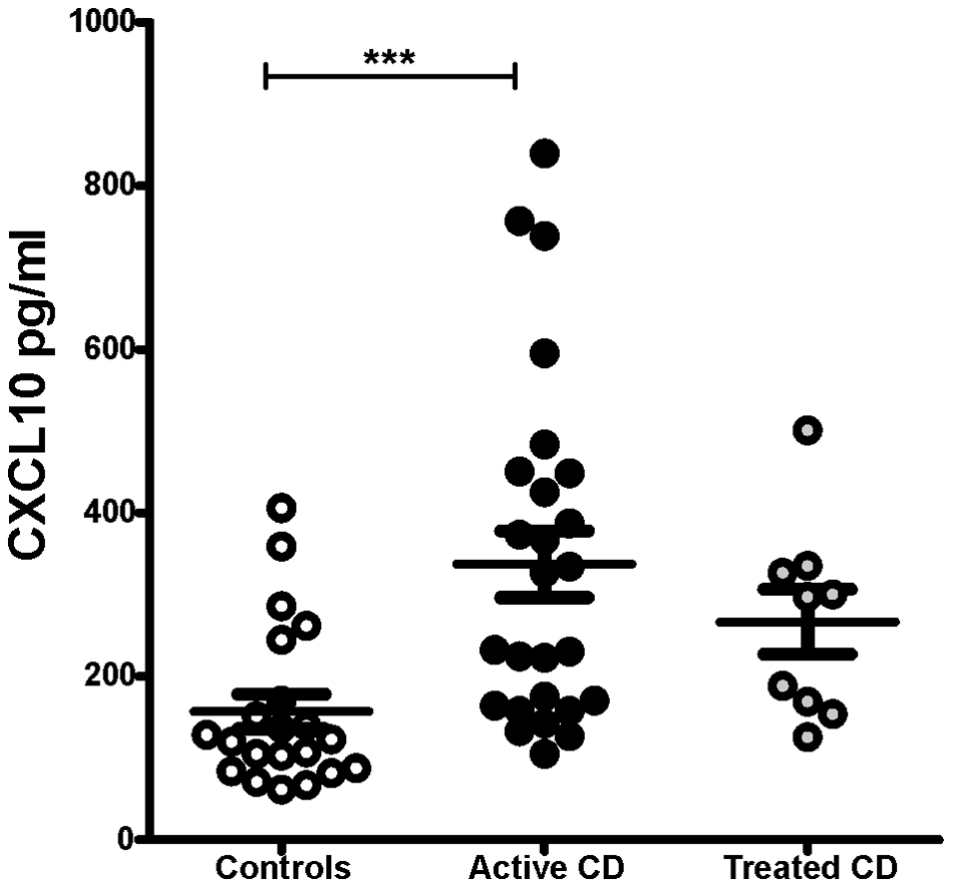
Serum levels of CXCL10. The CXCL10 concentrations in serum samples from 21 non-celiac individuals, 26 untreated celiac patients, and nine CD patients on a GFD were assessed by quantitative ELISA. The untreated CD patients presented higher levels of CXCL10 than the controls (unpaired t test; p = 0.0007). The treated celiac patients presented lower levels of CXCL10 than the untreated patients, although the difference was not statistically significant.

Increased levels of circulating CXCL10 have been detected in inflammatory processes and autoimmunity [15], [19]. However, to the best of our knowledge, no study has investigated CXCL10 expression in CD patients. To this end, we evaluated the levels of CXCL10 in serum samples from untreated CD patients, celiac patients on a gluten-free diet (GFD), and non-celiac controls using a quantitative ELISA. The concentration of CXCL10 was significantly higher in untreated CD patients than in non-CD controls. The levels of CXCL10 in the treated CD group were lower than those found in untreated patients, although this difference was not statistically significantly **(** **Figure 1** **)**. These results suggest a link between CXCL10 production and persistent gluten insult in untreated CD patients.

### Expression of CXCR3 Ligands (CXCL9, CXCL10, and CXCL11) in Duodenal Mucosa

**Figure 2:**
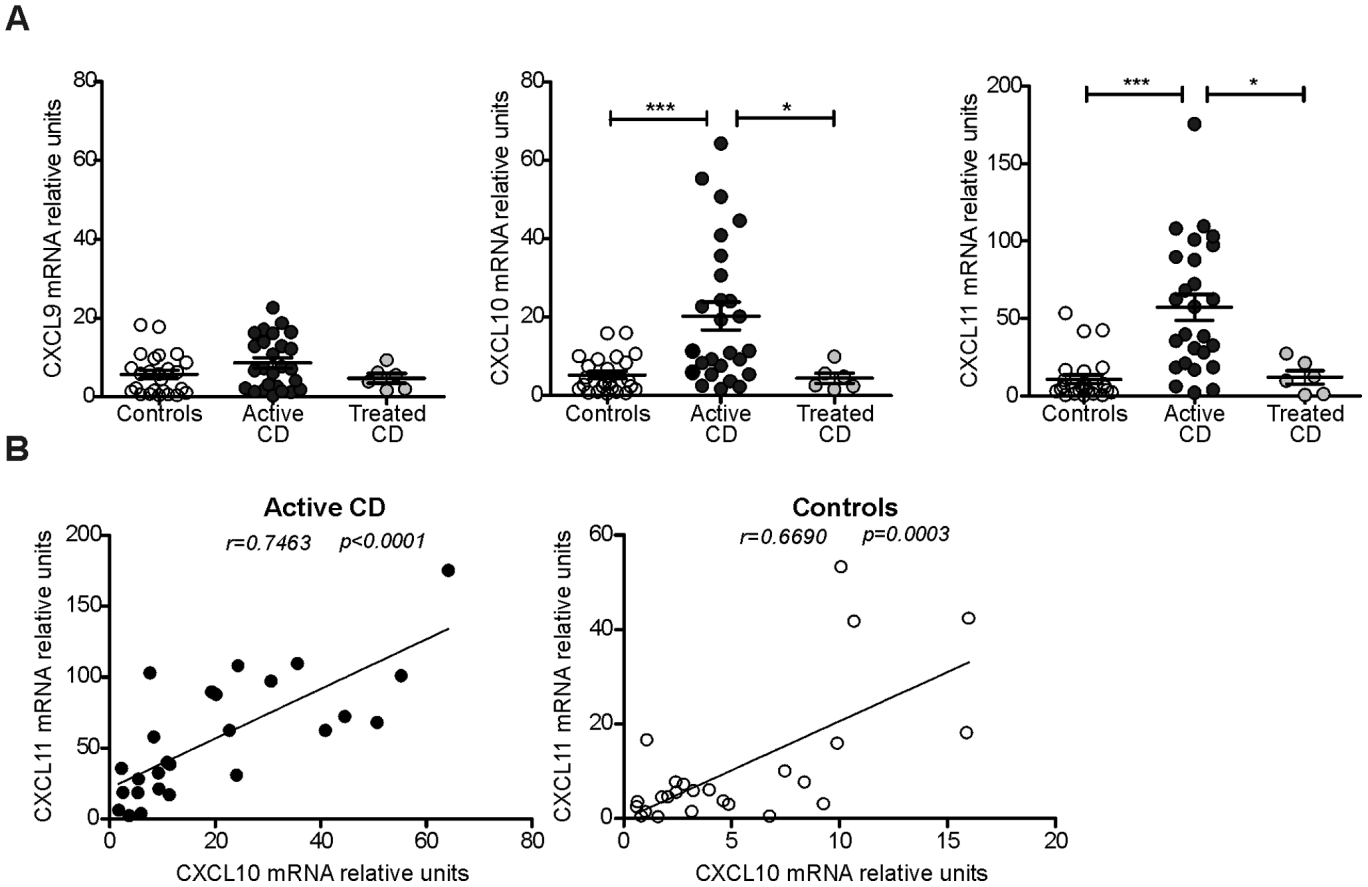
CXCL9, CXCL10, and CXCL11 mRNA levels in the duodenum. **a.** The mRNA expression levels of CXCR3 ligands were determined by real-time PCR. Duodenal biopsies from celiac individuals at the time of diagnosis (n = 26), celiac individuals on a GFD (n = 6), and non-CD controls (n = 25) were included. The untreated celiac patients expressed significantly higher levels of CXCL10 and CXCL11 than the treated patients (p = 0.0436 and p = 0.0160, respectively) and controls (p = 0.0002 and p<0.0001, respectively). There was no difference in the CXCL9 mRNA levels between the groups. The results are shown as relative units in reference with the levels of the housekeeping gene β-actin. An unpaired t-test was used to assess the significance of the differences. **b.** The correlation between the mRNA levels of CXCL10 and the mRNA levels of CXCL11 in duodenal samples from CD patients (black circles) and non-CD controls (white circles) was analysed. The CXCL10 and CXCL11 expression levels were correlated significantly in untreated CD patients (r = 0.7463, p<0.0001) and in non-CD controls (r = 0.6690, p = 0.0003).

The mRNA levels of CXCL9, CXCL10, and CXCL11 in duodenal tissues from untreated and treated CD patients and non-CD controls were assessed using quantitative PCR (**Figure 2a** **)**. Higher levels of CXCL10 and CXCL11 were obtained in the samples from untreated CD patients than in the control samples. In addition, patients on a GFD showed levels of these transcripts that were similar to those found in the controls. In contrast, no differences were observed in the CXCL9 mRNA levels between the three groups of samples. These findings indicate that CXCL10 and CXCL11 are actively produced in the duodenum of CD patients as a result of gluten intake, and a GFD effectively returns the activation of these genes to basal levels. Note that the mRNA expression levels of CXCL10 and CXLC11 exhibit a positive correlation in celiac and in control tissue samples (**Figure 2b**). CXCL9 was found to be expressed at markedly lower levels and independently of the other two chemokines (**data not shown**). These results suggest that CXCL10 and CXCL11 are produced as a consequence of gluten insult and that GFD deactivates the induction of these chemokines.

### Correlation between the Expression of CXCR3 Ligands and IFNγ, IFNβ, and TNFα in the Intestinal Mucosa

**Figure 3:**
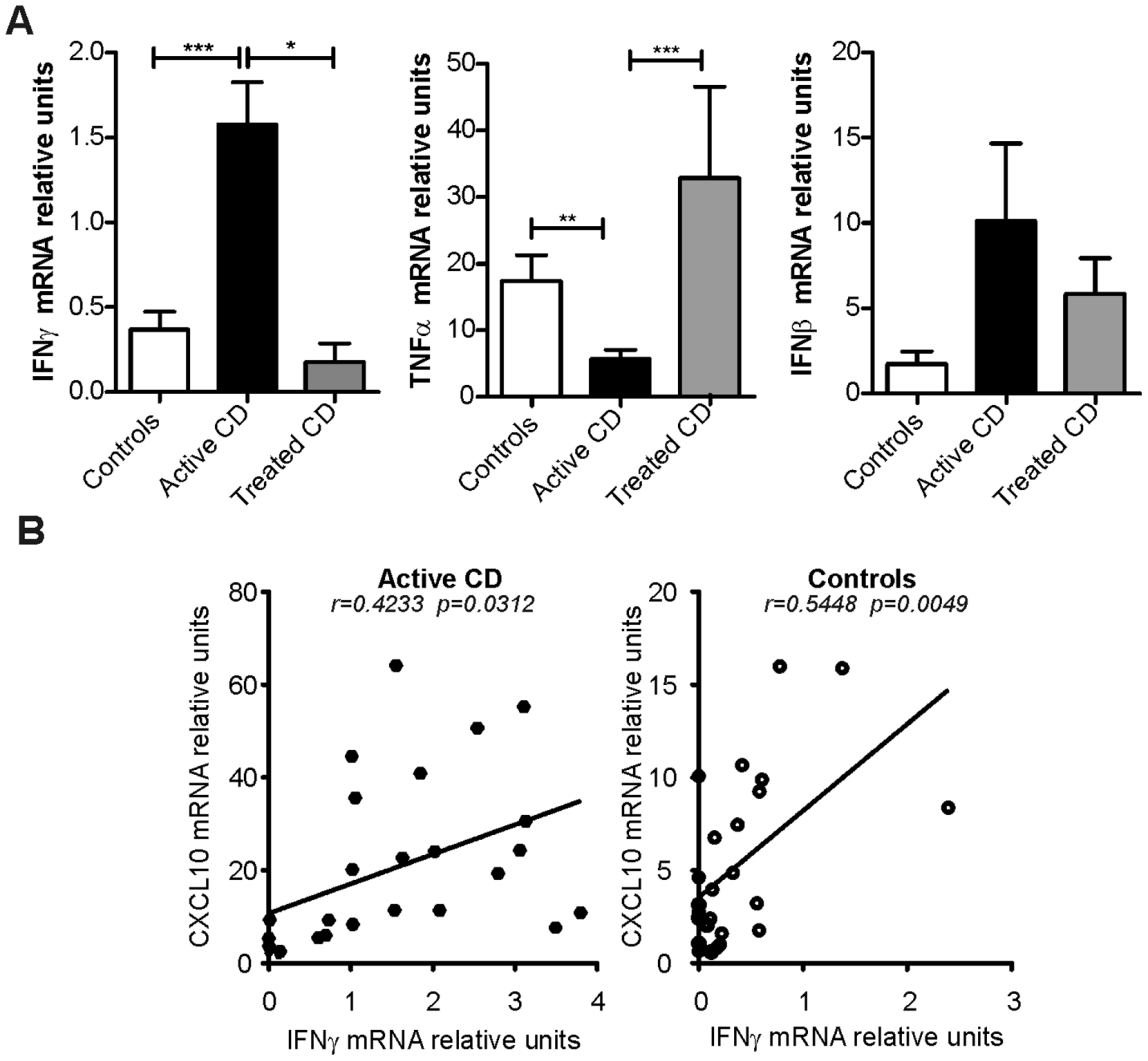
Expression of IFNγ, IFNβ, and TNFα in the duodenum and their correlation with the levels of CXCR3 ligands. **a.** The mRNA levels of IFNγ, IFNβ, and TNFα were determined by real-time PCR in the same set of samples that were previously tested: untreated celiac (n = 26), patients on a GFD (n = 6), and non-CD controls (n = 25). The IFNγ expression levels were significantly higher in the untreated celiac patients compared with the treated patients (p = 0.0123) and the controls (p<0.0001). The TNFα levels in the untreated celiac patients were lower than those found in the controls and the treated patients (unpaired t-test; p = 0.0054 and p = 0.0004, respectively). No significant difference was observed in the IFNβ expression levels between these groups. **b.** The correlation of the CXCL10 expression levels with the IFNγ levels in duodenal samples from untreated CD patients and non-CD controls was analysed. The IFNγ levels were positively correlated with the CXCL10 expression level in both untreated celiac patients (r = 04233, p = 0.0312) and non-CD controls (r = 0.5448, p = 0.0049). Linear regression analysis, Pearson’s coefficient, F-test.

The expression of CXCR3 ligands is modulated by different soluble factors, of which, IFNγ, IFNβ, and TNFα have a major influence [14]. To evaluate whether the expression levels of these inducers correlate with the levels of CXCR3 ligands, we determined the IFNγ, IFNβ, and TNFα mRNA levels in duodenal biopsies from untreated celiac patients, treated celiac patients, and non-celiac individuals. As expected, the IFNγ mRNA level was significantly increased in untreated patients. In contrast, the TNFα levels were lower in untreated CD patients compared with treated celiac patients and controls. However, the biopsies from untreated CD patients showed higher levels of IFNβ mRNA than those of the treated patients and controls, although the difference was not statistically significant (**Figure 3a**).

Remarkably, the CXCL10 mRNA levels were found to correlate positively with IFNγ expression in duodenal tissue from both CD patients and non-CD controls (**Figure 3b**), whereas CXCL10 expression did not correlate with the expression levels of either IFNβ or TNFα (**data not shown**). In contrast, the CXCL11 mRNA levels were correlated positively with IFNγ expression in untreated CD patients and with IFNβ in the control subjects (**Figure S1**). Consequently, CXCL10 showed a strong correlation with IFNγ expression in the intestinal mucosa in normal tissues and in severe enteropathy.

### Overproduction of CXCL10 in the Duodenal Mucosa of CD Patients

**Figure 4:**
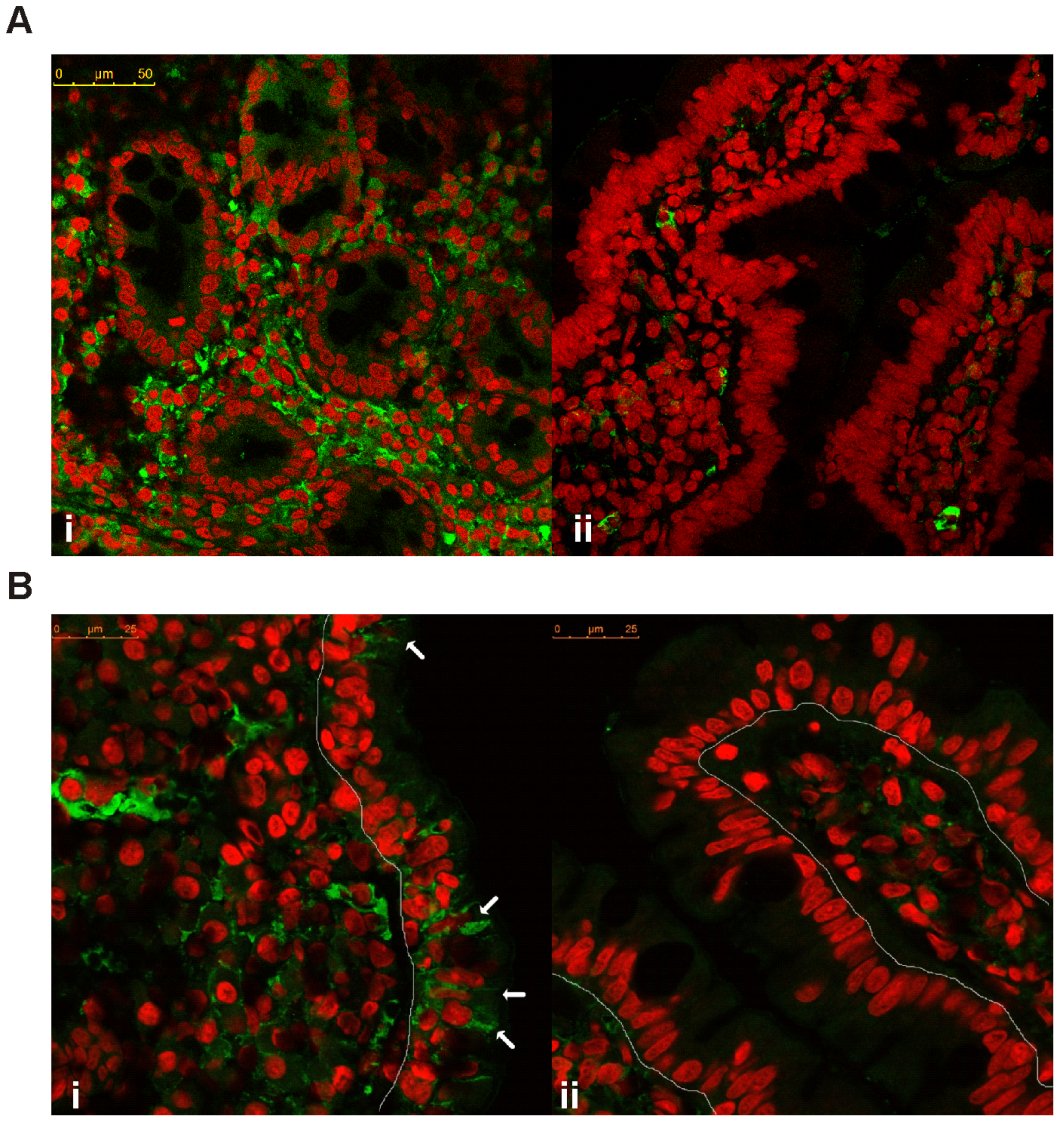
Overexpression of CXCL10 in the duodenal mucosa of untreated CD patients. A representative immunofluorescence confocal microscopic analysis of CXCL10 expression in duodenal sections of untreated CD (i) and control subjects (ii) is shown. **a.** Active CD patients showed a massive expression of CXCL10 in the entire mucosa, whereas the controls only showed isolated CXCL10^+^ cells in the LP. CXCL10 is shown in green, and the nuclei are shown in red. (Magnification, 630×). **b.** The arrows indicate epithelial cells that produce CXCL10 in the duodenum from an untreated CD patient (i). In non-CD controls (ii), CXCL10 expression was not observed in the epithelium, and CXCL10^+^ cells were rarely found in the LP. (Magnification, 1071×).

Immunofluorescence confocal microscopy analysis showed a massive production of CXCL10 in the duodenal mucosa of untreated CD patients (**Figure 4a**). Staining for CXCL10 appeared both inside LP cells and in the extracellular matrix. As a consequence of this overexpression, CXCL10^+^ cells could not be properly counted. In contrast, tissue sections from CD patients on a GFD or from non-CD controls showed CXCL10 staining only in a few cells in the LP. This massive expression of CXCL10 in the celiac duodenal mucosa correlates with the high CXCL10 concentration that is observed in the serum of untreated patients. Therefore, the small intestine of CD patients may be the primary source of this chemokine under gluten insult and could explain the high levels of circulating CXCL10 in untreated CD patients.

**Figure 5:**
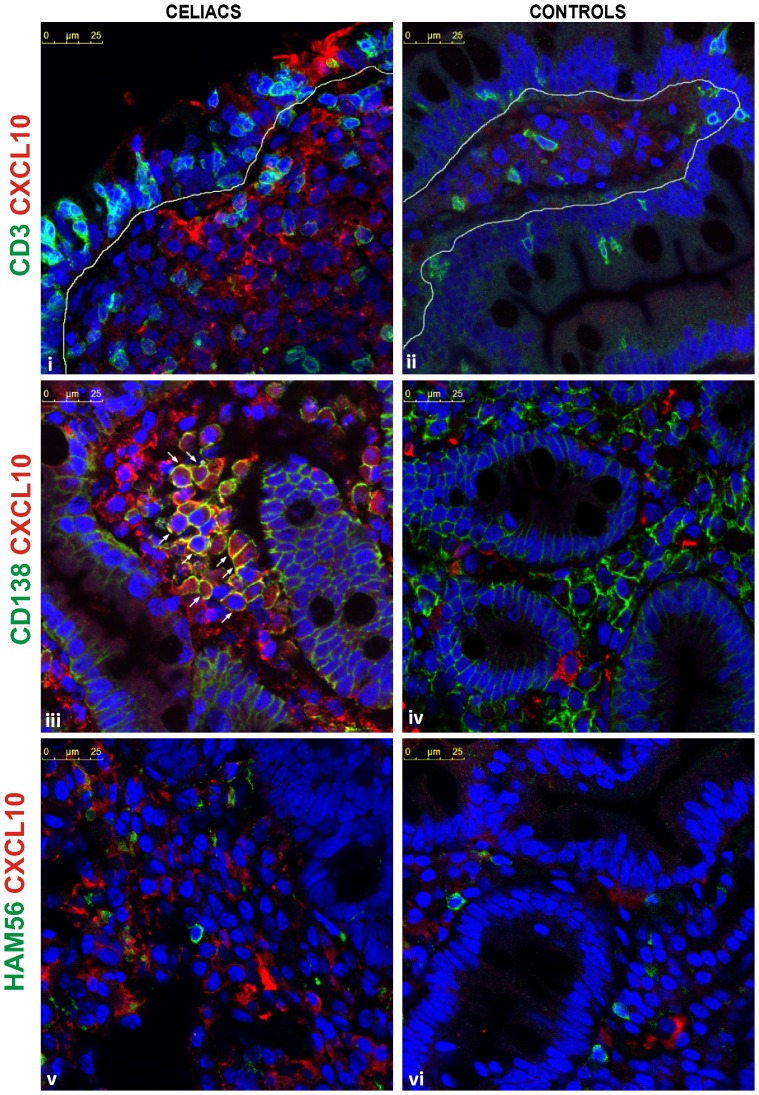
Cellular sources of CXCL10 in the small intestinal *lamina propria*. The cellular sources of CXCL10 in the duodenum were identified using immunofluorescence confocal microscopy. CD3^+^ cells were found to be negative for CXCL10 in both CD patients (i) and control subjects (ii). Numerous CD138^+^ cells that express CXCL10 were found in the celiac mucosa (iii). Plasma cells expressing CXCL10 were not found in the duodenum from non-CD controls (iv). HAM56^+^ cells did not produce CXCL10 in the celiac (v) and in the non-CD control intestinal mucosa (vi). CD3, CD138, and HAM56 are shown in green, CXCL10 is shown in red, and nuclei are shown in blue. (Magnification, 1071×).

Remarkably, the confocal microscopy analysis performed in this study also revealed that enterocytes from duodenal sections of untreated CD patients but not non-CD controls produce CXCL10 (**Figure 4b**). Because CD enteropathy is characterised by a massive infiltration of T lymphocytes and plasma cells, we further evaluated CD3^+^ and CD138^+^ cells as possible sources of CXCL10 production. Double staining for CD3 and CXCL10 showed no evidence that T lymphocytes are producers of this chemokine in celiac patients or in controls (**Figures 5i and 5ii**). As expected, the small intestine LP from untreated CD patients showed an increased number of plasma cells (CD138^+^ cells). Remarkably, a high proportion of CD138^+^ cells was found to be CXCL10-positive **(** **Figure 5iii** **)**. In contrast, a lower number of infiltrating plasma cells, which were all negative for CXCL10, was observed in the duodenum from non-CD controls **(** **Figure 5iv** **)**. The analysis of macrophages as putative CXCL10 producers revealed no evidence of HAM56^+^ CXCL10^+^ cells in the duodenal mucosa from celiac patients or controls **(** **Figures 5v and 5vi** **)**.

Altogether, these observations suggest that plasma cells are the main source of CXCL10 in the duodenum of untreated CD patients and also indicate that enterocytes play an important role as producers of this chemokine in the epithelial compartment.

### *In vitro* Analysis of CXCL10 Induction in Duodenal Tissue

**Figure 6:**
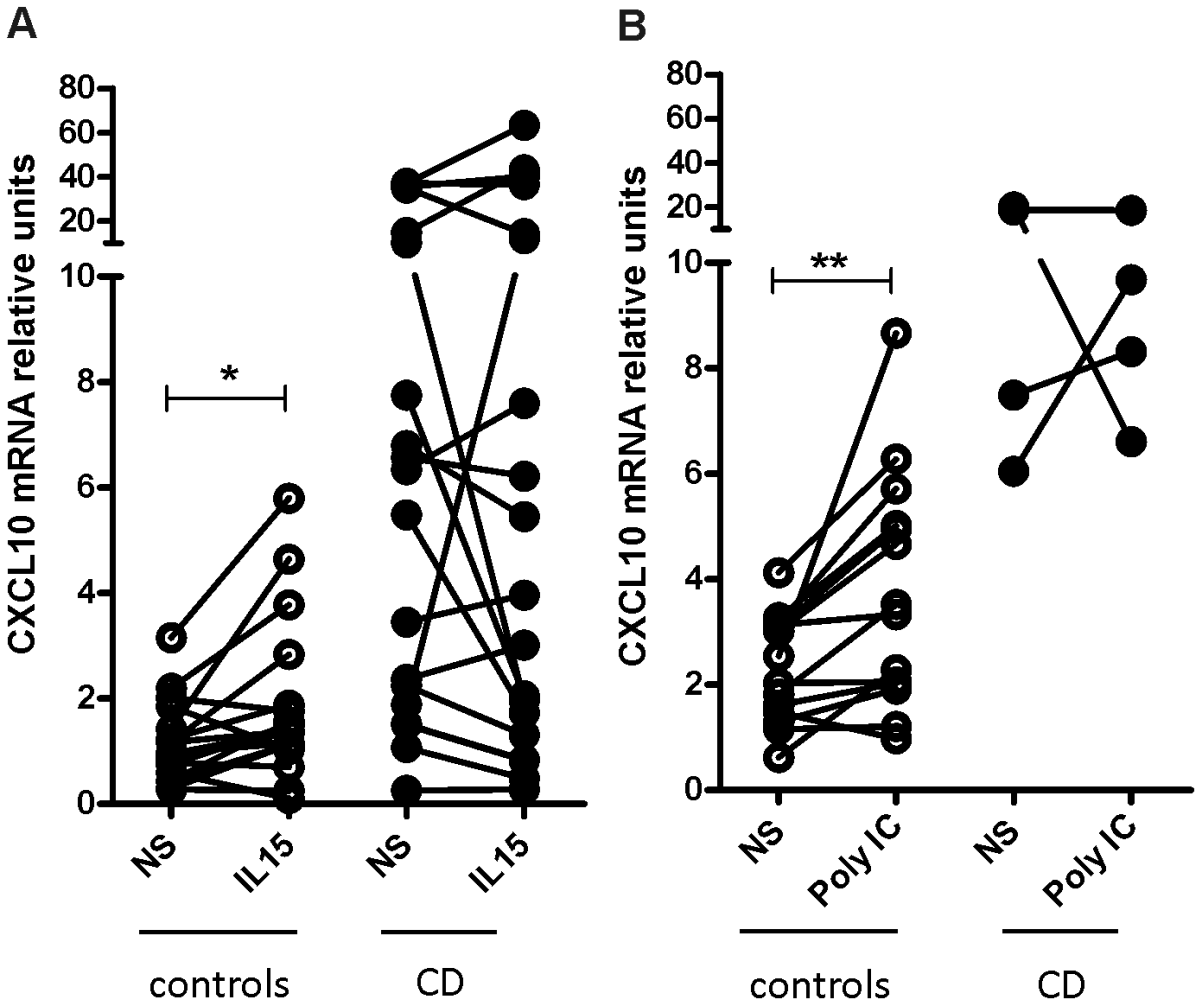
Induction of CXCL10 by IL-15 and poly I:C in the small intestine. Biopsies from celiac patients and non-CD controls were incubated for 3 h in the presence of IL-15 (**a**) or Poly I:C (**b**). A second biopsy from each patient was cultured with medium (NS). In non-CD controls, both IL-15 and poly I:C induced CXCL10 mRNA expression (p = 0.0100 and p = 0.0058, respectively; paired t-test). No significant changes were observed in the untreated CD group.

Viral infections (experimentally mimicked by poly I:C) and IL-15 have been suggested as critical triggers for the damage mechanisms that occur in the small intestinal mucosa during the early stages of CD [20]. Therefore, we aimed to evaluate whether IL-15 and poly I:C modulate the expression of CXCL10 in the small intestine. To this end, duodenal biopsies from untreated CD and non-CD controls were incubated in the presence or absence of IL-15 and poly I:C. Interestingly, both innate immunity stimuli caused a significant induction of CXCL10 expression in the controls but not in celiac individuals (**Figure 6**).

### Major Cell Populations Infiltrating the Small Intestinal Mucosa in Active CD Express CXCR3

Because the number of Th1 cells is characteristically increased in the duodenal mucosa in active CD and Th1 cells express CXCR3, we hypothesised that the overproduction of CXCL10 may be involved in Th1 cell recruitment to the intestinal mucosa in untreated CD. To this end, we analysed the presence of CXCR3^+^ cells in duodenal biopsies from CD patients and controls through confocal immunofluorescence microscopy and flow cytometry.

**Figure 7:**
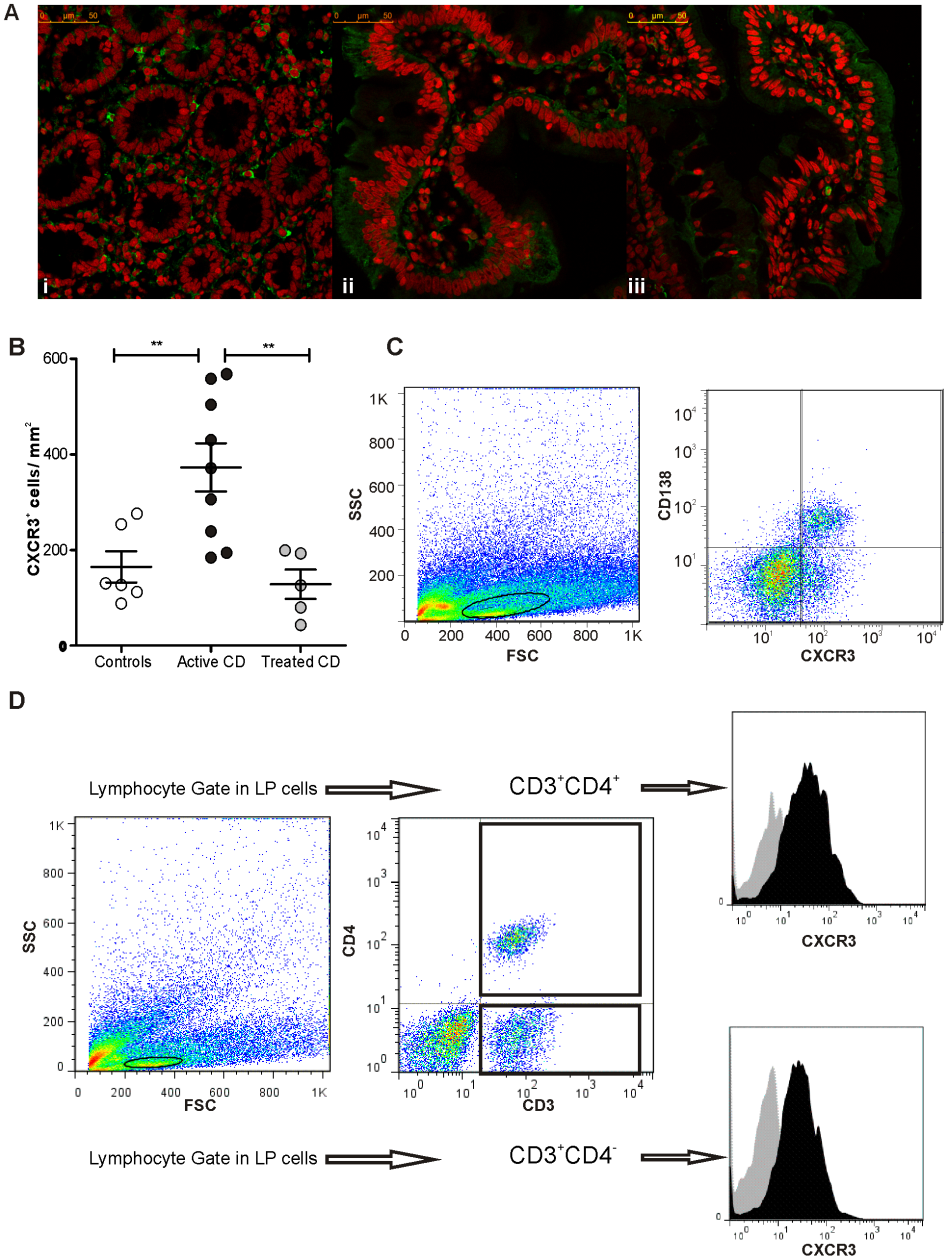
Infiltration of CXCR3^+^ cells in the *lamina propria* of small intestine mucosa. **a.** Confocal immunofluorescence for CXCR3 was performed in sections of duodenal biopsies from controls (i), untreated celiac patients (ii), and treated celiac patients (iii). CXCR3 is shown in green, and nuclei are shown in red. Untreated celiac patients showed a higher number of positive cells infiltrating the *LP*. (Magnification, 630×). **b.** The number of CXCR3^+^ cells in the LP was higher in the duodenal mucosa of untreated celiac patients (n = 9) compared with control individuals (n = 6) and treated patients (n = 5) (unpaired t-test; p = 0.0089 and p = 0.0055, respectively). The positive cells in LP regions from sections of duodenal biopsies were counted using immunofluorescence microscopy. **c.** Representative flow cytometric analysis from the LP compartment of a duodenal sample of an untreated CD patient showing plasma cells (CD138^+^) that express CXCR3. **d.** Representative flow cytometric analysis from the LP compartment of a duodenal sample of an untreated CD patient showing LP lymphocytes (CD3^+^ or CD4^+^) that express CXCR3.

Confocal microscopy studies showed an important infiltration of CXCR3^+^ cells in the LP of untreated CD patients **(** **Figure 7a** **)**. The counting of CXCR3^+^ cells in the LP regions of duodenal sections revealed an increased number of CXCR3^+^ cells in untreated CD patients compared with controls (**Figure 7b**). CD patients on a GFD presented numbers of CXCR3^+^ cells that were similar to those found in control subjects. These results suggest that gluten drives the active recruitment of CXCR3^+^ cells to the duodenal mucosa in untreated CD patients and that GFD restores the number of CXCR3^+^ cells to the baseline level that is found in healthy tissues.

T lymphocytes and plasma cells are the two main populations that infiltrate the LP in active CD. To evaluate whether this cell recruitment process is a consequence of the CXCR3/CXCL10 signalling axis, cells isolated from duodenum samples were subjected to flow cytometry analysis. LP CD3^+^ and CD4^+^ T lymphocytes and CD138^+^ plasma cells were found to express CXCR3 (**Figures 7c and 7d**). Altogether, these results highlight the relevance of CXCL10 in the recruitment of the major lymphocytic infiltrating populations in the LP of untreated CD patients.

### Intraepithelial Lymphocytes Express CXCR3

**Figure 8:**
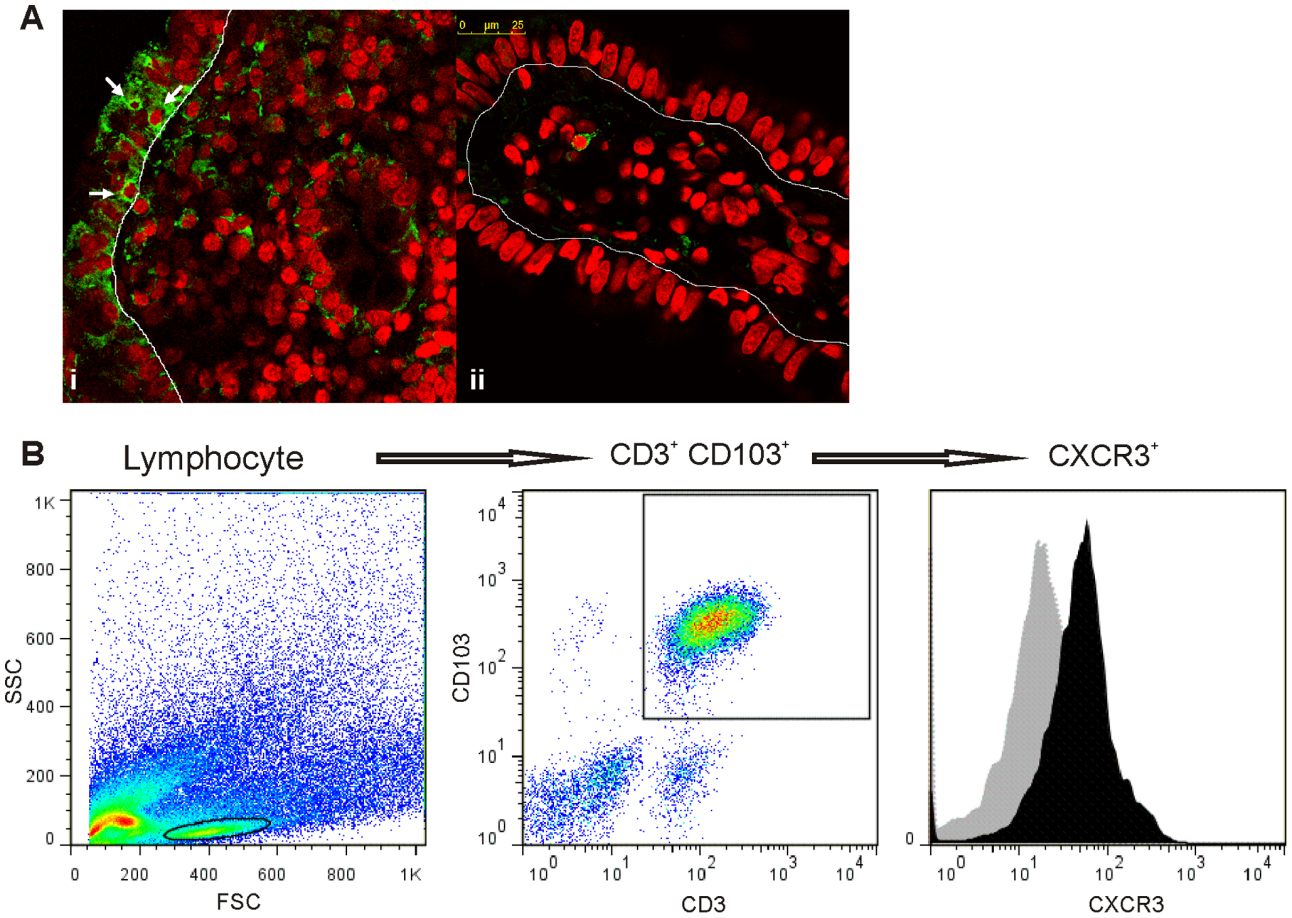
Infiltration of CXCR3^+^ cells in the intraepithelial compartment. **a.** Confocal immunofluorescence for CXCR3 was performed in sections of duodenal biopsies. (i) Intraepithelial lymphocyte CXCR3^+^ cells in untreated CD patients are indicated by arrows. (ii). CXCR3^+^ cells were rarely observed in the intraepithelial compartment in non-CD controls. The epithelium is delimited by a thin line. CXCR3 is shown in green, and nuclei are shown in red. (Magnifications, 630× (i) and 1071× (ii)). **b.** Representative flow cytometric analysis from the epithelial compartment of a duodenal sample of an untreated CD patient showing IELs (CD3^+^ CD103^+^) that express CXCR3.

Immunofluorescence confocal microscopy analysis showed CXCR3^+^ cells in the intraepithelial compartment. Characteristically, these CXCR3^+^ intraepithelial cells were detected more frequently in samples from untreated CD patients than in treated CD or non-CD controls **(** **Figure 8a** **)**. The analysis of the epithelial compartment by flow cytometry showed that CD3^+^ CD103^+^ IELs express CXCR3 **(** **Figure 8b** **)**. These findings suggest that the production of CXCL10 by the epithelium is responsible for the recruitment of CXCR3^+^ IELs, which are characteristically increased in untreated CD.

## Discussion

Gluten-specific CD4^+^ Th1 cells, which abundantly produce IFNγ, play a central role in tissue damage in CD enteropathy. Th1 cells and plasma cells are the two main populations that infiltrate the small intestine mucosa in untreated CD patients. In addition, CD8^+^ T cells, which may exert cytotoxic functions similarly to γ/δ T cells, are also expanded preferentially in the intraepithelial compartment of the celiac mucosa [1]. However, the mechanism through which immune cells arrive at the small intestine mucosa in CD enteropathy is not completely understood. Our results demonstrate that the CXCL10/CXCR3 chemokine axis plays a major role in immune cell recruitment in the inflamed intestinal mucosa of CD patients as a result of gluten insult.

The chemokine receptor CXCR3 is characteristically expressed in Th1 cells but is also highly expressed in innate lymphocytes, such as NK cells and NKT cells, and in cytotoxic T cell and B cells [12], [13], [21]. CXCR3 and its ligands (CXCL9, CXCL10, and CXCL11) are undoubtedly linked to the Th1 pattern and constitute an inflammatory pathway that coordinates the immune responses at sites of infection and inflammation. In activated T cells, CXCR3 expression is also important for the amplification of IFNγ-mediated recruitment into peripheral sites during infection and in autoimmunity [5].

CXCL10, CXCL9, and CXCL11 are differentially expressed in different pathological conditions, which suggests that these ligands have no redundant biological functions [14], [22]. In fact, different studies have revealed that CXCL10 plays a unique and important role in imprinting a pattern for the subsequent development of autoimmunity [23], [24]. In transgenic mice, the overexpression of CXCL10 in the pancreas induces a rapid recruitment of effector CD4^+^ and CD8^+^ T cells and accelerates the progression of type I diabetes [25]. Thus, CXCL10 can also act as a bystander effector that expands an autoaggressive immune response, which may result in autoimmune disease.

In agreement with findings in other chronic inflammatory conditions, such as type I diabetes [15], systemic sclerosis [19], and autoimmune thyroiditis [26], we found that the CXCL10 serum levels are significantly increased in untreated CD patients. Given the massive production of CXCL10 that was observed in the small intestine mucosa during active CD, we hypothesise that the small intestine may be the main source of circulating CXCL10 in untreated patients. In addition, the presence of high levels of this proinflammatory chemokine in untreated patients suggests that circulating CXCL10 can be considered a biomarker for CD. Further evaluation using many samples is required to determine the efficacy of this determination as a complementary test in the diagnosis and the follow-up of CD patients. Determination of CXCL9 and CXCL10 in serum also deserves further attention in order to evaluate whether serum levels of these chemokines reproduce the changes of expression observed in duodenal tissue.

We showed that CXCL10 is actively produced in the small intestine in untreated CD. Notably, the CXCL11 mRNA levels were also increased in this group. In contrast, changes in CXCL9 expression were not observed. A gluten-free diet reduced the levels of CXCL10 and CXCL11, which suggests that an active induction of these chemokines is a consequence of gluten insult. Furthermore, the expression of both chemokines showed a significant positive correlation in duodenal samples from CD patients and controls. Because CXCL10 and CXCL11 have similar promoter regions [14], these ligands may be induced by similar activation pathways. CXCL10 and CXCL11 are strongly induced by not only IFNγ but also type I IFNs and to a slight extent by TNFα [14]. To evaluate whether these cytokines are linked to the expression of the CXCR3 ligands in the small intestine mucosa, we assessed the expression of IFNγ, IFNβ, and TNFα in biopsy samples from untreated and treated CD patients and non-CD controls. As expected, higher IFNγ expression was observed in the small intestine of untreated CD patients. We showed a positive correlation between both CXCL10 and CXCL11 expression and the IFNγ levels in the duodenum of untreated CD patients, which suggests that IFNγ dominates the expression of these chemokines during chronic inflammation in CD. Altogether, these results suggest a differential regulation of CXCL9 in the duodenal mucosa and, to some extent, the existence of common regulatory pathways for CXCL10 and CXCL11 expression in the celiac mucosa. In addition, CXCL10 and CXCL11 exhibit different affinities to CXCR3 [14], [27], and although CXCL10 binds exclusively to CXCR3, CXCL11 binds to CXCR3 and also to CXCR7 [28]. These differences may indicate that these two chemokines have different functions. Further analysis is required to clarify the differential roles of CXCL10 and CXCL11 in the small intestine mucosa.

In agreement with the upregulation of CXCL10 that is observed at the mRNA level, a massive expression of CXCL10 protein was found in the intestinal mucosa from untreated CD patients. This pattern was due to a high number of CXCL10-producing cells and a large amount of the chemokine secreted into the extracellular space. Interestingly, the controls and treated patients only presented scarce CXCL10-producing cells in the LP. These findings indicate an active gluten-dependent production of CXCL10 in CD patients.

In different tissues, CXCL10 has been reported to be expressed by different cell types, such as CD4^+^ T cells, NK and NKT cells, macrophages, dendritic cells, fibroblasts, and endothelial and epithelial cells [7], [17], [29]. We then investigated the cellular sources of CXCL10 in the duodenal mucosa and identified CD138^+^ plasma cells as CXCL10-producing cells in the LP from untreated CD patients. In contrast, CD3^+^ (T cells) and HAM56^+^ (macrophages) did not express CXCL10. Considering the high number of infiltrating CD138^+^ cells in the LP in active CD, plasma cells would certainly be the main source of CXCL10 in the active disease. In addition, since CD138^+^ plasma cells express CXCR3, the CXCL10/CXCR3 chemokine axis would take a relevant role in the recruitment of plasma cells into the LP. Interestingly, CD138^+^ plasma cells produce locally anti-gliadin and anti-TG2 antibodies which play important roles in CD pathogenesis [30], [31].

Interestingly, a confocal immunofluorescence analysis showed that CXCL10 is also produced by enterocytes from the small intestine in active CD patients. CXCL10 staining was not observed in the epithelium of non-CD controls or treated CD patients. Therefore, enterocytes are active producers of CXCL10 in the duodenum of CD patients during the inflammatory process, and this production of CXCL10 is triggered by gluten intake. Although this study provides the first demonstration of the production of CXCL10 by enterocytes in the small intestine, similar results have been described in the colon [32], [33], and recently, the upregulation of CXCL10 was observed in colonic enterocytes in inflammatory bowel diseases [9]. Altogether, these findings indicate that enterocytes actively produce CXCL10 in a chronic inflammatory setting.

The mechanisms during the early phase of CD pathogenesis remain poorly understood. Viral infections have been suggested as inducers of an inflammatory cascade in the small intestine mucosa that can drive CD enteropathy in susceptible individuals [20]. In addition, IL-15 is another important player during the initial events. This cytokine participates in enterocyte damage by potentiating the cytotoxic activity of intraepithelial lymphocytes, among other effects [34]. Therefore, we aimed to evaluate whether poly I:C (an experimental model of viral infections) and IL-15 induce CXCL10 production in mucosal tissue. We found that both treatments induce a strong and rapid increase in CXCL10 expression in the intestinal mucosa of non-CD controls.

These results indicate that signalling pathways that are elicited by poly I:C and IL-15, which lead to the increased production of CXCL10, are operating fully in the small intestine mucosa. In contrast, CXCL10 induction was not observed in the duodenal samples from untreated CD patients. It is most likely that these pathways are already overactivated in active CD, and consequently, tissues with severe enteropathy cannot react to further stimulation.

Previous studies conducted by Lammers et al. [35] using immunohistochemistry showed the increased expression of CXCR3 in the small intestine mucosa in untreated CD patients. Importantly, in this work, confocal microscopy studies revealed a marked increase in the number of CXCR3^+^ cells in the LP from untreated CD patients. Disease remission, which was observed in patients on a GFD, is accompanied by a reduction in the number of CXCR3^+^ cells in the LP, and this decreased number is similar to the numbers found in non-CD controls. In addition, CXCR3^+^ intraepithelial cells were more frequently found in the mucosa from untreated celiac patients than in non-CD controls. Therefore, the finding that enterocytes produce CXCL10 during active CD may explain the characteristic increase in IELs that is observed in untreated CD. Flow cytometry analysis demonstrated that all cell populations that constitute the hallmark of CD enteropathy, e.g., plasma cells and T cells in the LP and IELs, express CXCR3.

To the best of our knowledge, this study provides the first demonstration of the expression of CXCR3 and its ligands in the small intestine. Our findings strongly suggest an active role for the CXCR3/CXCL10 axis in the pathogenesis of CD and a direct link between gluten ingestion and the activation of this axis. CXCL10 is actively produced in both the LP and the epithelium of the small intestine mucosa. Importantly, this chemokine was found to be also induced by proposed relevant innate stimuli in CD pathogenesis, including dsRNAs and IL-15 [20]. Consequently, the induction of CXCL10 may occur at two different stages of the disease. Initially, innate immunity activation causes CXCL10 induction, and this induction can be further increased by the presence of IFNγ during the chronic phase. In addition, we demonstrated that the major cell populations that infiltrate the small intestine mucosa in untreated CD express CXCR3. Therefore, the CXCR3/CXCL10 axis is not only involved in the recruitment of the critical cellular players responsible for mucosal damage in active CD, including Th1 cells and IELs, also may play a role in the initiation and perpetuation of the inflammatory process.

## Supporting Information

Figure S1 **The correlation of CXCL11 mRNA expression with IFNγ and IFNβ mRNA levels in duodenal samples from untreated CD patients and non-CD controls was analysed.** IFNγ was positively correlated with CXCL11 expression in untreated celiac patients (r = 0.5354, p = 0.0048) but not in non-CD controls (r = 0.0766, p = 0.7159). The analysis between IFNβ and CXCL11 expression in celiac patients did not show a significant correlation (r = 0.2945, p = 0.1442). In contrast, IFNβ was positively correlated with CXCL11 in the control group (r = 0.6061, p = 0.0013). Linear regression analysis, Pearson’s coefficient, F-test.
(TIF)
