# Supplementary figures and images for: Role of CXCR3/CXCL10 Axis in Immune Cell Recruitment into the Small Intestine in Celiac Disease
Source: PLoS One. 2014 Feb 20;9(2):e89068. doi: 10.1371/journal.pone.0089068 (PMC3930692; doi:10.1371/journal.pone.0089068)

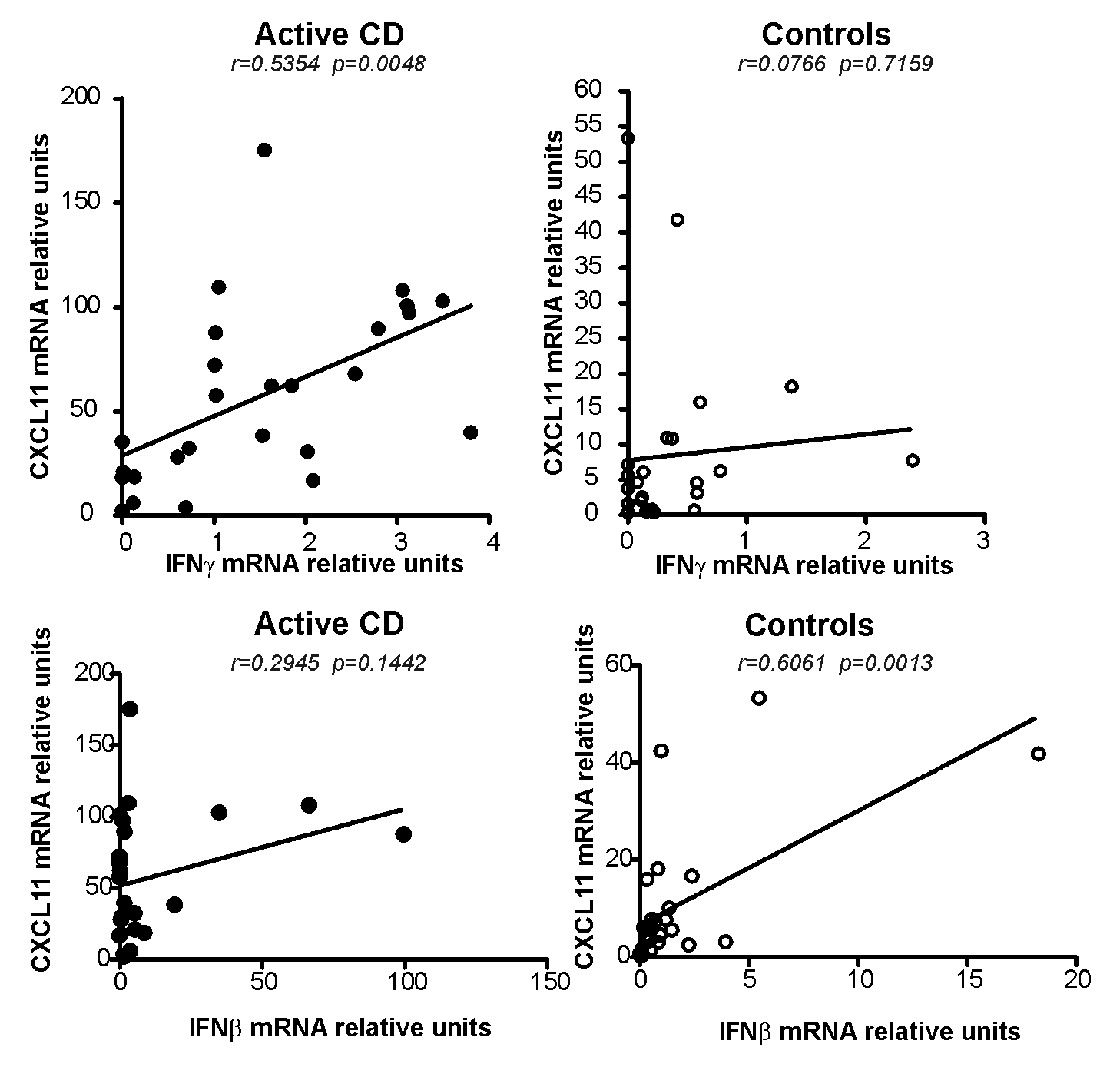

Supplement: Figure S1 — The correlation of CXCL11 mRNA expression with IFNγ and IFNβ mRNA levels in duodenal samples from untreated CD patients and non-CD controls was analysed. IFNγ was positively correlated with CXCL11 expression in untreated celiac patients (r = 0.5354, p = 0.0048) but not in non-CD controls (r = 0.0766, p = 0.7159). The analysis between IFNβ and CXCL11 expression in celiac patients did not show a significant correlation (r = 0.2945, p = 0.1442). In contrast, IFNβ was positively correlated with CXCL11 in the control group (r = 0.6061, p = 0.0013). Linear regression analysis, Pearson’s coefficient, F-test. (TIF) [file pone.0089068.s001.tif]
